# The nomograms for predicting overall and cancer-specific survival in elderly patients with early-stage lung cancer: A population-based study using SEER database

**DOI:** 10.3389/fpubh.2022.946299

**Published:** 2022-09-08

**Authors:** Gen Yu, Xiaozhu Liu, Yunhe Li, Yang Zhang, Ruxin Yan, Lingfeng Zhu, Zhongjian Wang

**Affiliations:** ^1^Department of Oncology, Ganxi Cancer Hospital, Pingxiang, Jiangxi, China; ^2^Department of Cardiology, The Second Affiliated Hospital of Chongqing Medical University, Chongqing, China; ^3^Department of Cardiothoracic Surgery, The Second Affiliated Hospital of Chongqing Medical University, Chongqing, China; ^4^College of Medical Informatics, Chongqing Medical University, Chongqing, China; ^5^Medical Data Science Academy, Chongqing Medical University, Chongqing, China; ^6^Department of Oncology, The First Affiliated Hospital of Army Medical University, Chongqing, China; ^7^Artificial Intelligence Laboratory, Pharnexcloud Digital Technology (Chengdu) Co., Ltd., Chengdu, China

**Keywords:** nomogram, elderly lung cancer, SEER, OS, CSS

## Abstract

**Purpose:**

Lung cancer is the leading cause of death from cancer and the number of operable elderly lung cancer patients is increasing, with advanced age being associated with a poorer prognosis. However, there is no easy and comprehensive prognostic assessment method for these patients.

**Methods:**

Clinicopathological data of patients aged 65 years or older with TNM stage I-II lung cancer from 2004 to 2018 were downloaded from the SEER database. Patients from 2004 to 2015 were randomized into a training group (*n* = 16,457) and a validation group (*n* = 7,048). Data from 2016 to 2018 (*n* = 6,231) were used for external validation. Two nomogram prognostic models were created after independent prognostic factors connected to both overall survival (OS) and cancer-specific survival (CSS) in the training set by using univariate and multivariate Cox proportional hazards regression analysis. In turn, overall survival (OS) and cancer-specific survival (CSS) were predicted for patients at 1, 3, and 5 years. Based on the concordance index (C-index), calibration curves, area under the receiver operating characteristics (ROC) curve (AUC), the time-dependent area under the ROC curve, the validity, accuracy, discrimination, predictive ability, and clinical utility of the models were evaluated. Decision curve analysis (DCA) was used to assess the clinical value of the models.

**Results:**

A total of 29,736 patients were included. Univariate and multivariate analyses suggested that age, race, gender, marriage, disease grade, AJCC stage, T-stage, surgery, radiotherapy, chemotherapy, and tumor size were independent risk factors for patient prognosis. These 11 variables were included in nomogram to predict OS and CSS of patients. C-indexes of OS for the training, validation and external validation sets were 0.730 (95% CI, 0.709–0.751), 0.734 (95% CI, 0.722–0.746), and 0.750 (95% CI, 0.734–0.766), respectively. The AUC results for the training and validation sets indicated good accuracy for this nomogram. The calibration curves demonstrated a high degree of concordance between actual and anticipated values, and the DCA demonstrated that the nomograms had better clinical application than the traditional TNM staging approach.

**Conclusion:**

This study identified risk factors for survival in operable elderly lung cancer patients and established a new column line graph for predicting OS and CSS in these patients. The model has good clinical application and can be a good clinical decision-making tool for physicians and patients.

## Introduction

Lung cancer is one of the most popular malignant tumors and the deadliest cancer, with a 5-year survival rate of ~16% worldwide (1). According to the most recent cancer statistics, it was estimated that 19,300,000 new tumor patients and over 10,000,000 deaths would occur in 2020 (1, 2). With increased awareness of medical screening and improved diagnostic techniques, the early detection rate of lung cancer is increasing, with the proportion of lung cancers presenting as early (operable) at the time of detection increasing from 25 to around 63% (3, 4).

The rate of systemic therapy in lung cancer patients over 65 years old was significantly lower than the rate of treatment in patients under 65 years old, according to a single institution study. Per the recent projections, lung cancer will increase significantly in patients over the age of 65 (5). This is why this study focuses on the elderly population. According to a recent study, elderly patients with lung cancer have substantially higher post-operative problems (26.0 vs. 13.3%) and mortality rate (8.2 vs. 2.2%) than younger patients after surgery (6). Even though surgery might achieve successful resection of the tumor, about half of early-stage lung cancer will recur after surgery, which may lead to death (7). Thus, proper selection of surgery candidates would contribute to an increase in life quality and a decrease in morbidity. To choose surgical patients with a better prognosis, it is advantageous to develop a clinically appropriate and straightforward grading system. Notable heterogeneity exists among patients with early lung cancer in terms of demographic and clinicopathological data, including age, sex, T and N stages, pathological type, tumor stage, and applied therapy strategies. Thus, the prognosis of early lung cancer varies significantly between patients with different characteristics. Adjuvant therapy for patients who have undergone surgery for early lung cancer should be categorized into distinct prognosis groups.

In the United States, nearly 70% of lung cancer cases and >70% of lung cancer deaths occur in patients over the age of 65 (8). However, older adults are underrepresented in clinical trials and making treatment decisions in this population is challenging (9). In general, the available data to guide decision-making in older people is limited. Our study focused on older lung cancer patients aged ≥65.

SEER indicates Surveillance, Epidemiology, and End Results database. Approximately 35% of the U.S. population is covered by this database, which pertain to cancer prognosis. Nomograms provide personalized risk estimates by combining and showing significant prognostic criteria, and they are superior than other existing decision aids for more precisely predicting cancer patient outcomes (10). We used the SEER database to build and verify a web-based model for predicting the survival of elderly patients with early lung cancer, which may be useful for prognostic prediction, treatment strategy selection, and follow-up management of these patients.

## Materials and methods

### Patients and methods

The SEER^*^stat software (version 8.3.5; http://seer.cancer.gov/seerstat/) was used to extract all patient data from the National Cancer Institute's SEER database. The National Cancer Institute (NCI) sponsors the SEER database, which collects statistics on cancer incidence and outcomes.

Clinicopathological information on early-stage (operable) elderly lung cancer patients from 2004 to 2018 was selected. Institutional review and informed permission were not necessary for this investigation because no patients were directly involved and no personal identifiable information from the SEER data was used.

Inclusion criteria were as follows: (1) Site recode ICD-10 codes: lung and bronchus (C34.0–C34.3, C34.8, and C34.9); (2) Age ≥65; (3) Known survival time.

**Figure 1:**
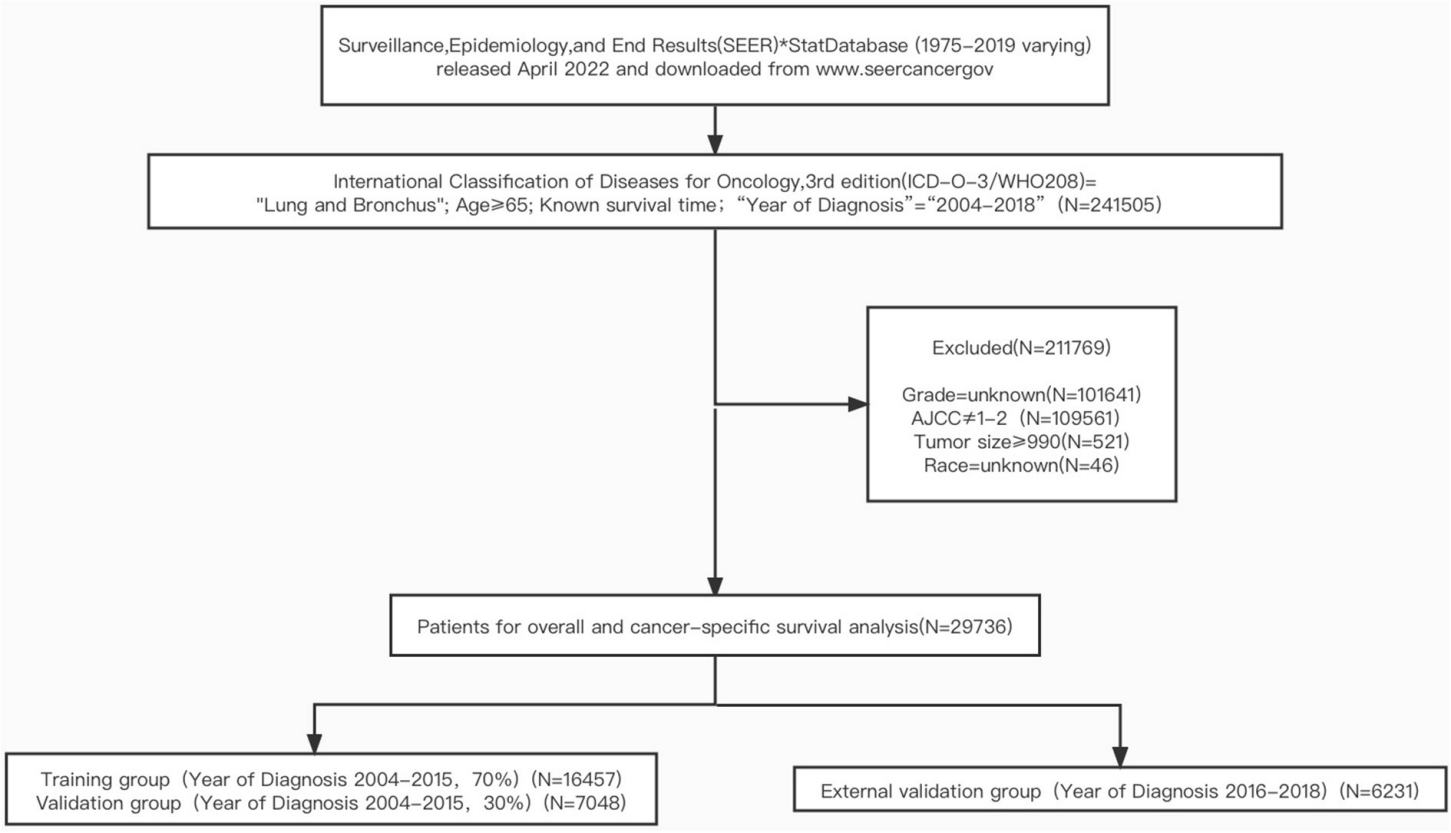
The flowchart of including and dividing patients.

The exclusion criteria were as follows: (1) unknown histological grade; (2) AJCC stage IV; (3) Tumor size ≥990; (4) unknown race; (5) Patients with incomplete or unclear data on other indicators. The patient selection process is presented in Figure 1.

### Study variables and outcomes

A total of 12 indicators were analyzed in this study, from the clinical and pathological characteristics of the patients, variables included in the study included age (65–69, 70–74, 75–79, 80–84, ≥85), race (black, white, other races), sex (female, male), marital status (married, unmarried), disease classification (grade I, II, III, IV), AJCC stage (IA/IB, IIA/IIB, IIIA/IIIB), T (T1, T2, T3), N (N0, N1), surgery (no, yes), radiotherapy (no, yes), chemotherapy (no, yes), tumor size (<5, 5–10, and ≥10 cm).

### Variable correction

Reclassification of T-stage, N-stage and M-stage recorded in the SEER database according to the 8th edition of the American Joint Committee on Cancer Staging manual (11).

### Endpoint definition

Primary endpoint one was OS, defined as time from randomization to death due to any cause. Primary endpoint two was CSS, or to say lung cancer-specific survival (LCSS), defined as the time from randomization to death as a result of lung cancer. In addition, the survival rates at 1, 3, and 5 years were examined. The C-index, receiver operating characteristics (ROC) curve, time-dependent area under the ROC curve (AUC), decision curve, and calibration curve were used to evaluate the nomogram's validity, accuracy, discrimination, predictive capacity, and clinical value. In this study, patients who had been alive at the time of their last follow-up were omitted from the data.

### Statistical analysis

We randomly assigned 70% (*n* = 16,457) of patients from 2004 to 2015 to the training cohort and 30% (*n* = 7,048) of patients from 2004 to 2015 to the validation cohort for nomogram construction and validation. The external validation cohort included 6,231 patients from the SEER database from 2016 to 2018.

Various Cox proportional hazard regression models were used to examine the impact of potential predictors on both overall survival (OS) and cancer-specific survival (CSS). For OS and CSS, the univariate Cox proportional hazard regression model contained 12 potential factors. A backward stepwise technique was used to enter variables with a univariable *p* < 0.05 into the multivariable model and assess their significance. The Cox proportional hazards regression technique was used to obtain hazard ratios (HRs) and confidence intervals (CIs).

A nomogram was created based on the data from the training cohort, and its ability to predict outcomes was examined in the validation cohort. R software (version 3.4.4) was used to build an effective prognostic nomogram for the training cohort based on variables that were statistically significant in the multivariate analysis. Using a scale ranging from 0 to 100, each variable's score was determined. When all variables were taken into account, each patient's overall score was calculated. Finally, each patient's OS and CSS probabilities at 1, 3, and 5 years were estimated.

Based on bootstrapped calibration curves and a C-index, the nomogram-based prediction model was verified. In order to test the final model's discrimination abilities, we used the ROC curve approach. In this investigation, a bootstrap technique with 1,000 resamples was used to create calibration curves to compare observed and projected survival. A decision curve analysis was performed to evaluate the clinical benefit of our model.

We used the survival package surv_cutpoint() function in R, of which the basic principle is based on log-rank test by using Kaplan-Meier curve to take the best cutoff value of the total risk score of the training set column line graph: 168.4566. The best cutoff value was greater than the cutoff value for the high-risk group, and less than the cutoff value for the low-risk group. To compare patient survival between groups, we used Kaplan–Meier curves and log-rank testing. We ran univariate and multivariate Cox proportional hazards regression analyses with SPSS software (version 24.0). We created the nomogram, C-indices, ROCs, calibration curves, DCA curves and Kaplan–Meier curves using R software (version 4.0.2) and relevant packages (“rms,” “DynNom,” “nomogramFormula,” “survival,” “foreign,” “survivalROC,” “ggDCA,” “survminer”). Using the X-Tile software, we calculated the cutoff value (version 3.6.1). Statistical significance was defined as a two-sided *p* < 0.05.

## Results

### Basic characteristics

The selected patients were all >65 years old. In the training and validation groups, most of the patients were white (84.87%), and had a tumor in early T stage (96.01%) and N stage (88.64%) and with early histological staging (61.98%). In terms of gender and marital status, no significant difference was detected between these two groups. Most patients had undergone surgery (76.03%) and a small number of patients had received radiotherapy (21.42%) and chemotherapy (16.81%). In the external validation group, the picture was largely consistent: The majority of the patients (84.10%) were white, with tumors in the early T stage (90.08%) and N stage (92.33%), as well as early histological staging (67.87%). There was no discernible difference between the two groups in terms of gender or marital status. More than half of the patients (62.88%) underwent surgery and a few patients received radiotherapy (31.76%) and chemotherapy (15.10%) (Table 1). The distribution of pathogenic features and clinical information did not differ significantly across the training and validation sets, as shown in Table 2 (all *p* > 0.05).

**Table 1:** Clinicopathological characteristics of elderly lung cancer patients—the training group and the validation group.

**Characteristic**	**Overall**	**Training set**	**Validation set**	***p*-value**
**N**	**23,505**	**16,457**	**7,048**	
**Age (%)**
65–70	6,005 (25.55)	4,149 (25.21)	1,856 (26.33)	0.4161
70–75	6,271 (26.68)	4,397 (26.72)	1,874 (26.59)	
75–80	5,841 (24.85)	4,099 (24.91)	1,742 (24.72)	
80–85	3,734 (15.89)	2,641 (16.05)	1,093 (15.51)	
≥85	1,654 (7.04)	1,171 (7.12)	483 (6.85)	
**Race (%)**
Black	1,741 (7.41)	1,185 (7.20)	556 (7.89)	0.1737
White	19,948 (84.87)	13,992 (85.02)	5,956 (84.51)	
Other	1,816 (7.73)	1,280 (7.78)	536 (7.60)	
**Sex (%)**
Female	11,852 (50.42)	8,323 (50.57)	3,529 (50.07)	0.4884
Male	11,653 (49.58)	8,134 (49.43)	3,519 (49.93)	
**Marital status (%)**
Married	12,752 (54.25)	8,917 (54.18)	3,835 (54.41)	0.7576
No	10,753 (45.75)	7,540 (45.82)	3,213 (45.59)	
**Grade (%)**
I	4,164 (17.72)	2,949 (17.92)	1,215 (17.24)	0.1204
II	10,403 (44.26)	7,303 (44.38)	3,100 (43.98)	
III	8,263 (35.15)	5,717 (34.74)	2,546 (36.12)	
IV	675 (2.87)	488 (2.97)	187 (2.65)	
**AJCC (%)**
I	19,896 (84.65)	13,948 (84.75)	5,948 (84.39)	0.4936
II	3,609 (15.35)	2,509 (15.25)	1,100 (15.61)	
**T (%)**
T1	12,138 (51.64)	8,489 (51.58)	3,649 (51.77)	0.4955
T2	10,429 (44.37)	7,326 (44.52)	3,103 (44.03)	
T3	938 (3.99)	642 (3.90)	296 (4.20)	
***N*** **(%)**
N0	20,834 (88.64)	14,590 (88.66)	6,244 (88.59)	0.9072
N1	2,671 (11.36)	1,867 (11.34)	804 (11.41)	
**Surg (%)**
No	5,635 (23.97)	3,943 (23.96)	1,692 (24.01)	0.9511
Yes	17,870 (76.03)	12,514 (76.04)	5,356 (75.99)	
**Radiation (%)**
No	18,471 (78.58)	12,943 (78.65)	5,528 (78.43)	0.7273
Yes	5,034 (21.42)	3,514 (21.35)	1,520 (21.57)	
**Chemotherapy (%)**
No	19,554 (83.19)	13,734 (83.45)	5,820 (82.58)	0.1033
Yes	3,951 (16.81)	2,723 (16.55)	1,228 (17.42)	
**Tumor size (%)**
<5 cm	20,646 (87.84)	14,459 (87.86)	6,187 (87.78)	0.9611
5–10 cm	2,579 (10.97)	1,804 (10.96)	775 (11.00)	
≥10 cm	280 (1.19)	194 (1.18)	86 (1.22)	

**Table 2:** Clinicopathological characteristics of elderly lung cancer patients——the external validation.

**Characteristic**	**Overall**
	***N*** **=** **6,231**
**Age (%)**
65–70	1,654 (26.54)
70–75	1,811 (29.06)
75–80	1,444 (23.17)
80–85	854 (13.71)
≥85	468 (7.51)
**Race (%)**
Black	499 (8.01)
White	5,240 (84.10)
Other	492 (7.90)
**Sex (%)**
Female	3,358 (53.89)
Male	2,873 (46.11)
**Marital status (%)**
Married	3,301 (52.98)
No	2,930 (47.02)
**Grade (%)**
I	1,414 (22.69)
II	2,815 (45.18)
III	1,920 (30.81)
IV	82 (1.32)
**AJCC (%)**
I	4,836 (77.61)
II	1,395 (22.39)
**T (%)**
T1	3,538 (56.78)
T2	2,075 (33.30)
T3	618 (9.92)
***N*** **(%)**
N0	5,753 (92.33)
N1	478 (7.67)
**Surg (%)**
No	2,313 (37.12)
Yes	3,918 (62.88)
**Radiation (%)**
No	4,252 (68.24)
Yes	1,979 (31.76)
**Chemotherapy (%)**
No	5,290 (84.90)
Yes	941 (15.10)
**Tumor size (%)**
<5 cm	5,667 (90.95)
5–10 cm	530 (8.51)
≥10 cm	34 (0.55)

### Univariate and multivariate cox regression analysis

To identify predictors of OS and CSS among the 16,457 patients comprising the training cohort, univariate and multivariate analyses were performed. As can be seen in Table 3, age, race, gender, marriage, disease grade, AJCC stage, T-stage, surgery, radiotherapy, chemotherapy, and tumor size were independent risk factors affecting patient prognosis. The Cox proportional hazards regression model was utilized to investigate in depth the effects of various parameters. OS and CSS multivariate analysis revealed increased hazard ratios (HRs) for the following characteristics: older age, male gender, unmarried, higher histology grade and T stage, no surgery of the primary tumor, larger tumor sizes, and having received radiotherapy or chemotherapy (*p* < 0.05).

**Table 3:** Univariate and multivariate analyses of OS and CSS in training set.

**Variable**	**Univariate analysis**		**Multivariate analysis**	
	**HR (95%CI)**	***p*-value**	**HR (95%CI)**	***p*-value**
**Age**
65–70	1		1	
70–75	1.23 (1.16–1.3)	<0.001	1.18 (1.12–1.25)	<0.001
75–80	1.58 (1.49–1.67)	<0.001	1.46 (1.38–1.54)	<0.001
80–85	2.01 (1.89–2.13)	<0.001	1.68 (1.58–1.79)	<0.001
≥85	2.84 (2.63–3.06)	<0.001	1.89 (1.75–2.05)	<0.001
**Race**
Black	1		1	
White	0.99 (0.92–1.07)	0.815	1.08 (1–1.16)	0.048
Other	0.77 (0.7–0.85)	<0.001	0.85 (0.77–0.94)	0.0014
**Sex**
Female	1		1	
Male	1.37 (1.32–1.42)	<0.001	1.38 (1.32–1.43)	<0.001
**Marital status**
Married	1		1	
No	1.19 (1.14–1.23)	<0.001	1.19 (1.14–1.24)	<0.001
**Grade**
I	1		1	
II	1.43 (1.35–1.51)	<0.001	1.4 (1.33–1.49)	<0.001
III	1.89 (1.79–2.01)	<0.001	1.6 (1.51–1.7)	<0.001
IV	2.08 (1.86–2.32)	<0.001	1.69 (1.51–1.89)	<0.001
**AJCC**
I	1		1	
II	1.67 (1.6–1.76)	<0.001	1.53 (1.44–1.62)	<0.001
**T**
T1	1		1	
T2	1.42 (1.37–1.48)	<0.001	1.29 (1.24–1.35)	<0.001
T3	2.33 (2.13–2.55)	<0.001	1.37 (1.23–1.52)	<0.001
**Surg**
No	1		1	
Yes	0.33 (0.32–0.35)	<0.001	0.3 (0.28–0.32)	<0.001
**Radiation**
No	1		1	
Yes	2.09 (2–2.18)	<0.001	0.72 (0.68–0.77)	<0.001
**Chemotherapy**
No	1		1	
Yes	1.18 (1.12–1.24)	<0.001	0.83 (0.78–0.88)	<0.001
**Tumor size**
<5 cm	1		1	
5–10 cm	1.7 (1.61–1.8)	<0.001	1.24 (1.16–1.31)	<0.001
≥10 cm	2.24 (1.92–2.61)	<0.001	1.75 (1.49–2.05)	<0.001

### Prognostic nomograms for OS and CSS

**Figure 2:**
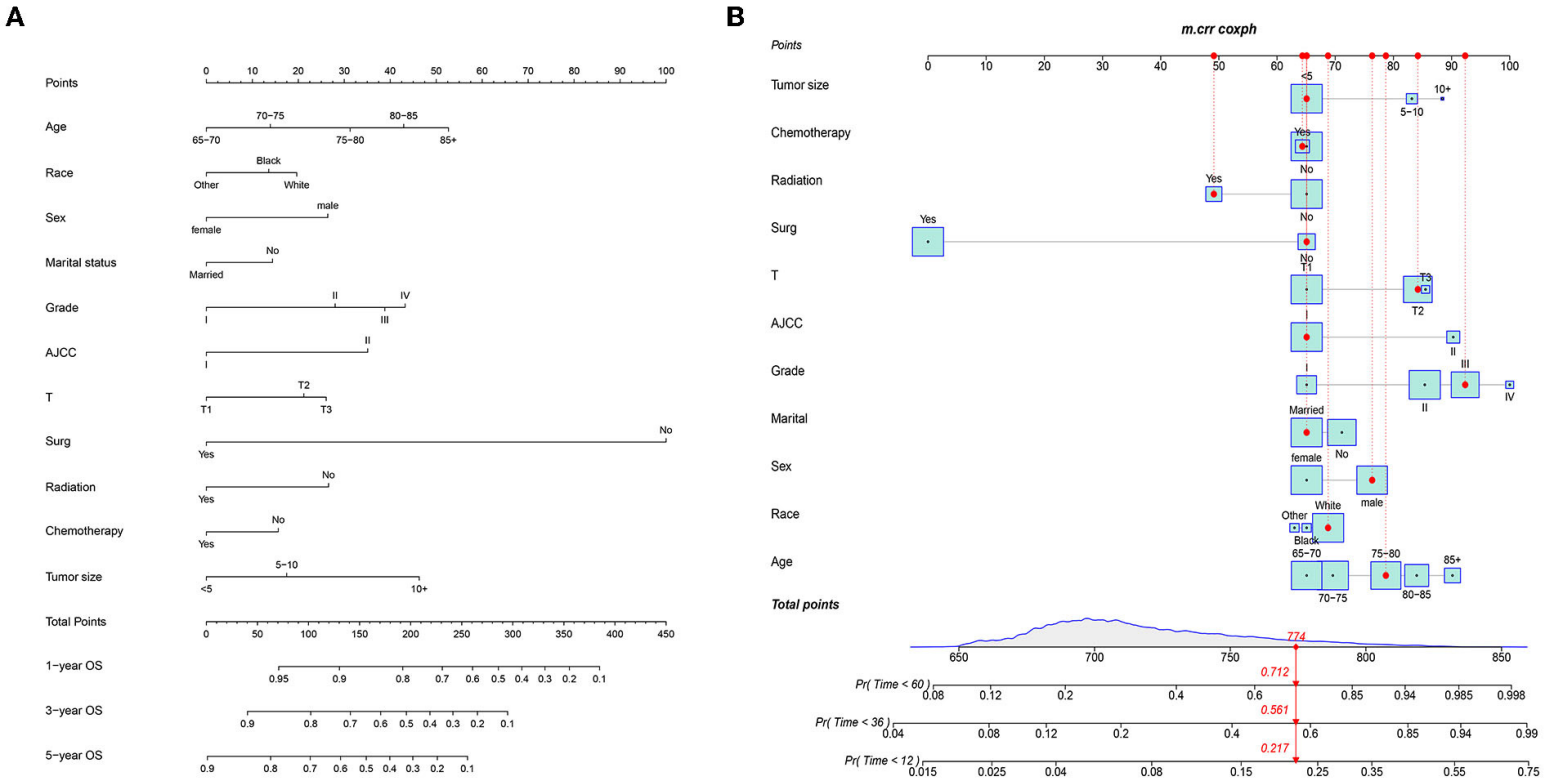
Prognostic nomograms of 1-, 3-, and 5-year OS **(A)** and CSS **(B)**.

The prognostic nomogram comprised all significant independent factors of the Cox proportional hazards regression in the training group. Figure 2A depicts the OS nomogram for the first, third, and fifth years, while Figure 2B depicts the CSS nomogram for the first, third, and fifth years. By combining the scores associated with each characteristic and projecting the total scores to the bottom scale, it is possible to estimate the likelihood of OS and CSS at 1, 3, and 5 years. Our model can be used to predict the outcomes of individual patients according to their characteristics.

### Confirmation of the nomograms

The C-index of the OS predictive model was 0.730 (95% CI, 0.709–0.751) in the training group and 0.734 (95% CI, 0.722–0.746) in the validation group. In external validation, the C-index was 0.750 (95% CI, 0.734–0.766), indicating good discrimination. As for the CSS nomogram, the C-index for the training group was 0.755, 0.714, and 0.689 for 1, 3, and 5 years respectively. In the validation group, the C-indexes for 1, 3, and 5 years were 0.754, 0.718, and 0.69 respectively. Patients in the external validation group had a C-index of 0.781 for 1 year only, as the longest CSS was 35 months.

**Figure 3:**
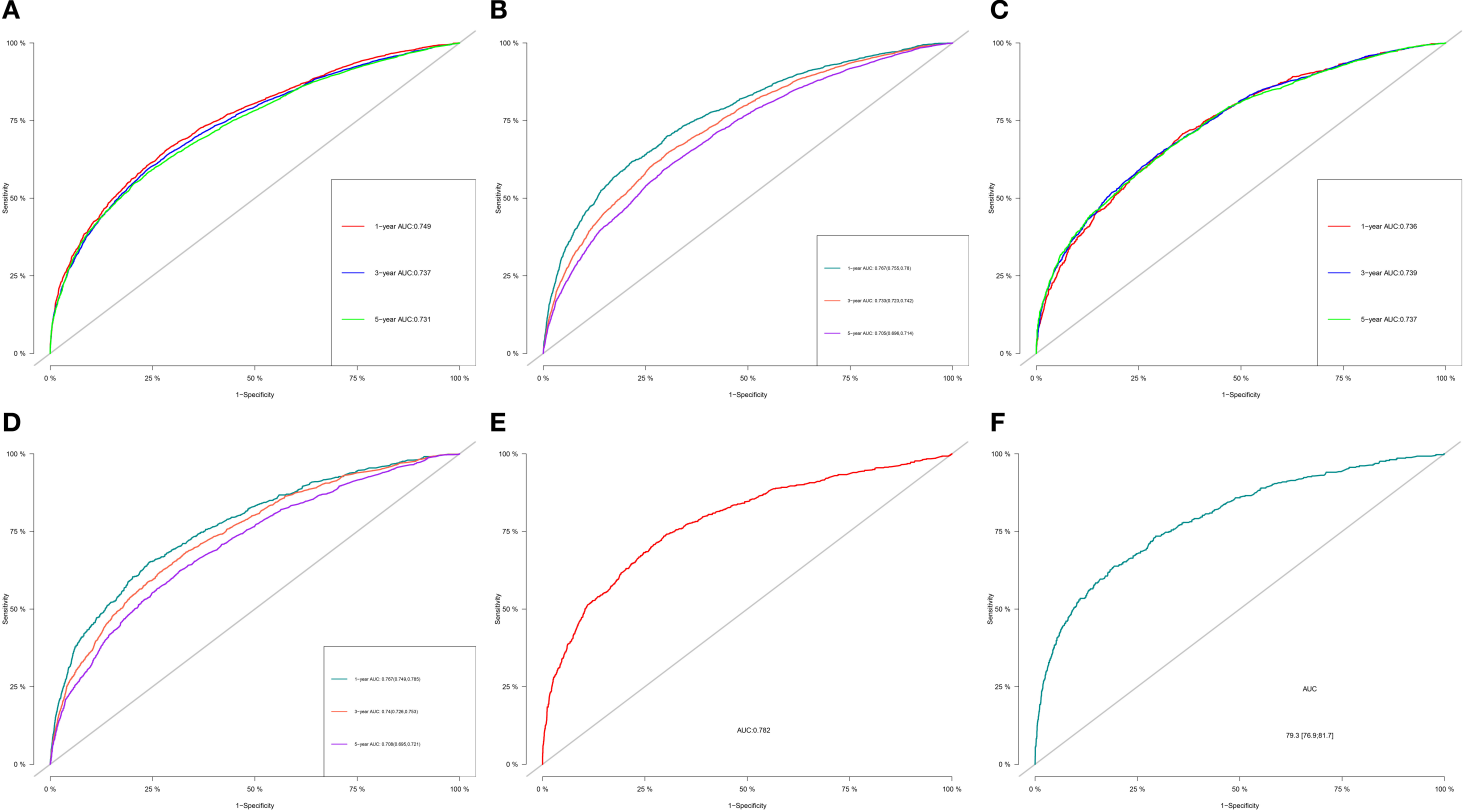
1-, 3-, and 5-year ROC curves of OS and CSS in training group **(A,B)**, validation group **(C,D)** and external validation group **(E,F)** for validating nomogram models.

**Figure 4:**
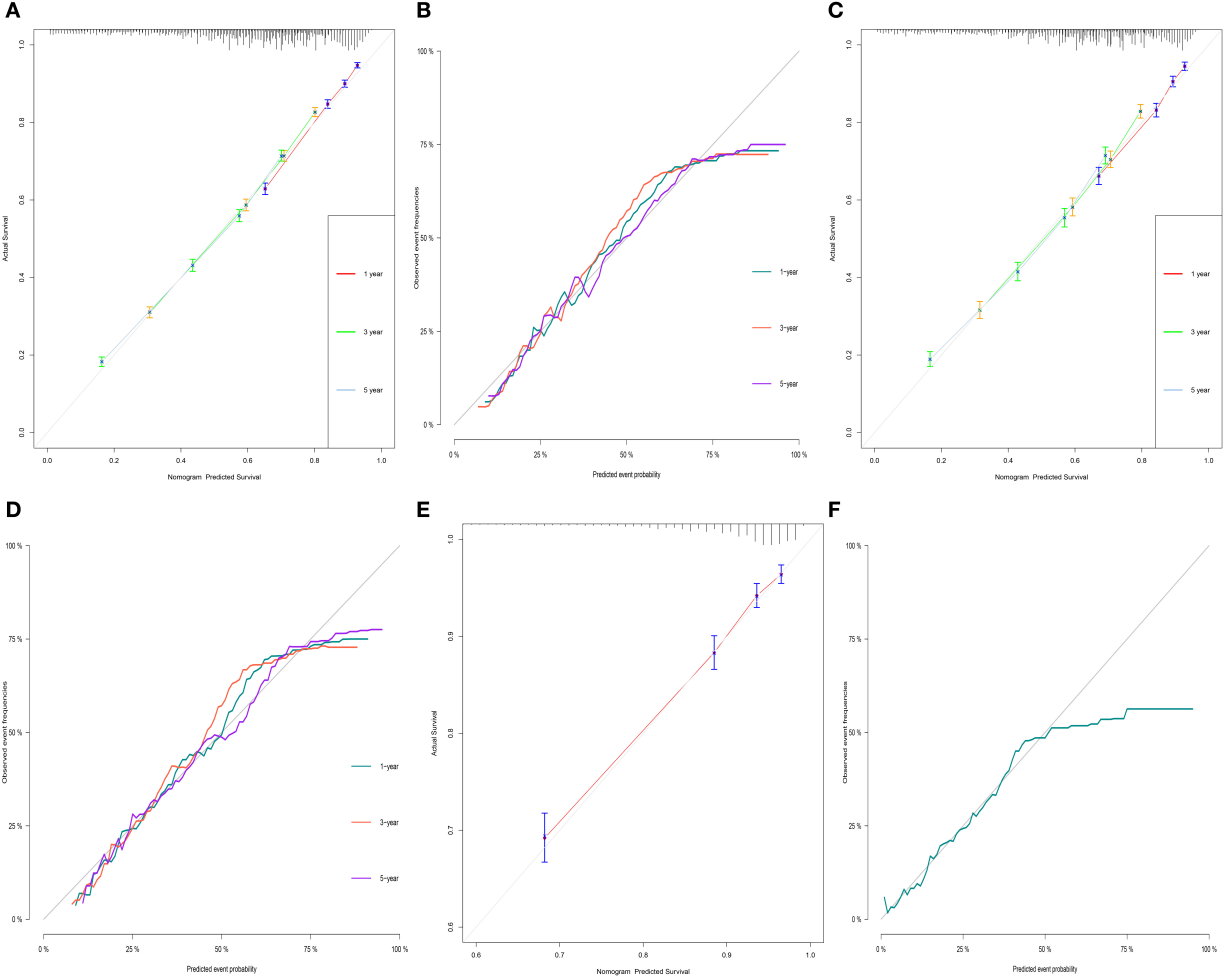
Calibration curves of OS and CSS in training group **(A,B)**, validation group **(C,D)** and external validation group **(E,F)** for validating nomogram models.

For OS, the 1-, 3-, and 5-year AUCs were 0.749, 0.737 and 0.731 for the training group, 0.736, 0.739, and 0.737 for the validation group and 0.782 for the external validation group (1 year only). As to the CSS nomogram, the 1-, 3-, and 5-year AUCs were 0.767 (95% CI, 0.755–0.78), 0.733 (95% CI, 0.723–0.742), and 0.705 (95% CI, 0.696–0.714) for the training group and 0.767 (95% CI, 0.749–0.785), 0.74 (95% CI, 0.726–0.753), and 0.708 (95% CI, 0.695–0.721) for the validation group and 0.793 (95% CI, 0.769–0.817) for the external validation group (1 year only; Figure 3). These results suggested the predictive nomograms were with good discrimination performance. Furthermore, calibration curves for 1,−3-, and 5-year indicated a good consistency between the observed survival and the predicted survival in both in OS and CSS (Figure 4).

**Figure 5:**
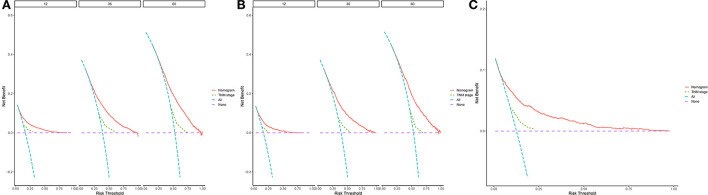
1-, 3-, and 5-year decision curve analysis of nomogram. **(A)** The DCA curves of nomogram in training group. **(B)** The DCA curves of nomogram in internal validation group. **(C)** The DCA curves of nomogram in external group. The net benefit is shown by the *y*-axis, and the threshold probability is represented by the *x*-axis. No patients have died on the purple line, while all patients have died on the blue line. The net benefit of the model exceeds all deaths or no deaths when the threshold probability is between 20 and 60%.

Figure 5 depicts the DCA curves for the prognostic nomogram and TNM staging scheme. DCA revealed that the prognostic nomogram had greater net advantages than the TNM staging approach, indicating greater clinical application value.

### Risk classification system

**Figure 6:**
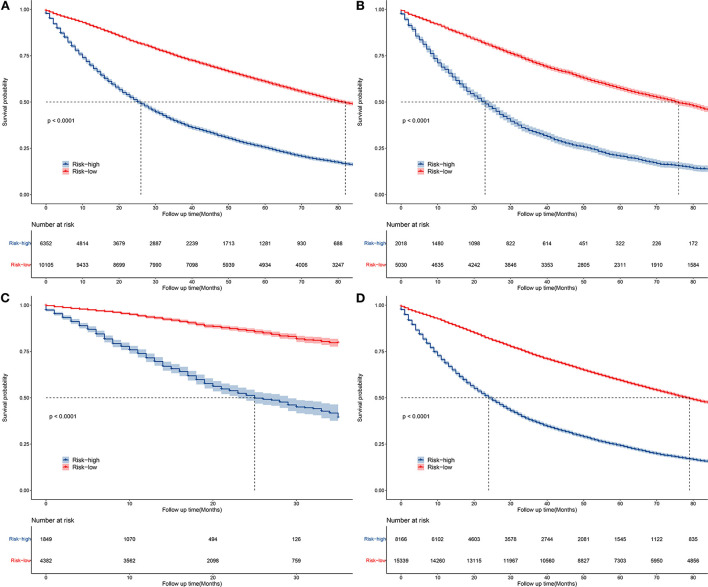
Kaplan–Meier curves of OS for patients in the low-, and high-risk groups in the training group **(A)**, validation group **(B)**, external validation group **(C)**, and general group **(D)**.

According to the overall score generated by the prognostic nomogram, all cases were split into two subgroups, each representing a different prognosis. Figure 6 depicts the Kaplan-Meier survival curve that indicated the prognosis for each subgroup. Based on OS events, in the training set, the high-risk group had a 1-year OS of 69.9%, a 3-year OS of 39.2% and a 5-year OS of 39.2%. In the low-risk group, the 1-year OS was 91.4%, the 3-year OS was 74.6%, and the 5-year OS was 60.9%. In the validation set, the high-risk group had a 1-year OS of 67.7%, a 3-year OS of 34.0%, and a 5-year OS of 20.7%. The low-risk group had a 1-year OS of 90.0%, a 3-year OS of 71.5%, and a 5-year OS of 57.3%. Overall, the high-risk group had a 1-year OS of 68.8%, a 3-year OS of 37.5%, and a 5-year OS of 24.1%. The low-risk group had a 1-year OS of 90.9%, a 3-year OS of 73.4%, and a 5-year OS of 59.3%. In the external validation set, the maximum survival time in the source data was 35 months, with a minimum of 71.6% 1-year OS in the high-risk group and 93.6% 1-year OS in the low-risk group. There were statistically significant differences in survival outcomes between the two groups.

## Discussion

The baseline demographic and clinical characteristics of the patients were analyzed, Then, a prognostic nomogram for the 1-, 3-, and 5-year OS and CSS of elderly patients with early lung cancer were built and validated, which could be useful for prognostic evaluation, treatment strategy selection, and follow-up management. The prognostic nomogram had superior prediction accuracy for lung cancer than the present TNM staging system, according to the ROC, DCA, and error curves. Furthermore, the OS nomogram was qualified to split lung cancer patients into low and high risk categories, implying that this nomogram might be routinely used to predict lung cancer patients' prognosis.

In the present population-based cohort study, univariate and multifactorial analyses revealed that age, race, gender, marriage, disease grade, AJCC stage, T-stage, surgery, radiotherapy, chemotherapy, and tumor size were all independent prognostic predictors for older patients with early-stage lung cancer. Similar to a large number of previous studies, the univariate and multifactorial COX regression analyses in this study found higher risk rates for patients with characteristics such as advanced age, male, unmarried, late histological stage, and large T-stage. In addition, inoperative primary tumor predicted a worse prognosis, suggesting that although older patients usually have a higher surgical risk secondary to their age and other underlying diseases commonly seen in the elderlies, postoperative morbidity and mortality are acceptable even in older patients (12, 13). After accurate and thorough preoperative assessment and preparation, the choice of an appropriate pneumonectomy is safe in elderly patients with lung cancer after accurate and thorough preoperative assessment and preparation, such as minimally invasive surgery (14).

Advanced age is consistently one of the risk factors affecting the long-term survival of oncology patients (15), and a variety of factors can help explain this phenomenon. The worst prognosis in older patients is associated with reduced physiological reserve, reduced effectiveness of cancer treatment, and increased risk of toxic side effects and death (16). Patients with non-small cell lung cancer (NSCLC) over 80 years of age were reported to be less likely to receive chemotherapy as initial treatment than patients aged 70–79 years (12.3% vs. 40.9%) (17). It is also important to note that unlike older patients, the development of early-stage lung cancer (lung cancer occurring before the age of 45 years) is primarily associated with genetic factors (18). Some studies have suggested that targeted genomic alterations are significantly increased in younger patients and targeted therapy is associated with improved survival (19). The present study found that advanced age negatively affected not only OS but also CSS of patients, which is relevant and consistent with the above-mentioned reasons.

Epidemiological studies have shown that men have a poorer prognosis than women in a wide range of cancer types not related to reproductive function (20), including but not limited to lung, liver and melanoma (21). A pooled analysis of five previous randomized trials showed that women with lung cancer had higher response rates and longer survival to chemotherapy compared to men with lung cancer, and that differences in OS persisted after adjustment for age, stage, performance status and histology (22). A study of 2,724 men and 1,894 women with lung cancer conducted in the USA showed that the risk of death following a diagnosis of lung cancer was significantly higher in men than in women (adjusted relative risk: 1.20, 95% CI: 1.11–1.30) (23). One of the possible explanation for this difference between men and women is smoking, as smoking rate is higher among men than women, and there are also gender differences in susceptibility to tobacco carcinogens (24). In addition, it has been suggested that an important reason for the poorer prognosis in men is that men showed increased endogenous and induced DNA damage as well as higher levels of unrepaired DNA than females (25). This study was conducted in older patients with more cumulative mutations (26), and perhaps the increased DNA damage would be more pronounced and worth further exploration.

Interestingly, this study showed a relatively poor prognosis for patients who had received radiotherapy or chemotherapy, since patient who have received radical radiotherapy or radical chemotherapy usually do not undergo further surgery, and the treatments are mutually exclusive. Patients, who opt for relatively conservative treatments including radiotherapy and chemotherapy, instead of surgery, fail to have the tumor load eradicated, which leads to a poorer prognosis. Guo et al. concluded that for elderly patients with LA-NSCLC, the curative-intent treatment (surgery or CRT) conferred better survival compared to chemotherapy alone, RT alone and BSC (27). We validated this conclusion with a larger sample size.

The most controversial finding was whether N stage was included as a factor independently affecting prognosis: in our study, N stage had no significant effect on prognosis, in contrast to the findings of Liang et al. (28) in NSCLC, who found that a greater number of ELNs is associated with more-accurate node staging and better long-term survival of resected NSCLC. However, the study did not distinguish between patient staging and age. The fact that N stage was not included In the present research as an independent risk factor for prognosis in elderly patients with early-stage lung cancer is an unconventional but novel perspective, and we believe the most important reason for this is as follow: we analyzed patients with N0 or N1 lung cancer in this study, N1 nodes include ipsilateral intrapulmonary, peribronchial, hilar lymph nodes, and direct invasion of the primary tumor, which does not influence treatment decisions requiring surgery, nor even the specific options for non-surgical therapies such as radiotherapy and targeted and immunotherapy, and so have limited impact on prognosis.

The primary tumor staging system, TNM staging, is crucial for assessing the prognosis of cancer (29). However, TNM has significant limitations: first, patients with the same TNM stage but different survival outcomes are forced into the same disease stage, introducing heterogeneity. Second, TNM staging system does include tumor, lymph nodes, or metastases as continuous variables. It is also important to note that the TNM system does not incorporate many key variables, although in general, the prognosis will be worse when the TNM stage is too high. It does not incorporate other variables associated with prognosis, such as histology, treatment status, gene mutation status, etc (30). Hence, a more comprehensive and precise prognostic model is needed. In contrast, the nomogram is a major innovation in assessing prognosis and can incorporate numerous variables that will hopefully help patients and physicians in all aspects of decision making. The prognostic nomogram demonstrated more lung cancer prediction accuracy in this study than the current TNM staging approach, according to the DCA.

The AUC values for these nomograms are 0.73–0.75, which are not very high, and we considered that it is related to the heterogeneity of tumors in elderly patients. However, the results of C-index, calibration curve and DCA, except for the AUC values, are relatively balanced indicating the good performance of the present model. The inclusion of basic patient information, clinical consensus factors known to be associated with lung cancer prognosis, and common treatments in our column nomograms allows for individualized patient assessment and the most accurate prediction of cancer patient outcomes possible. The column line graph prognostic model can greatly facilitate risk stratification and treatment planning, as well as more precise inclusion criteria for clinical trials, and also help with patient counseling and follow-up.

To visualize and integrate these independent risk factors, the present study constructed a column line graph to calculate an intuitive, quantitative, individual probability of survival for older patients with early-stage lung cancer. After internal validation, the model showed good discriminatory power and net clinical benefit to assist in treatment decision making for older patients with early-stage lung cancer. All factors listed in the column line graphs are common clinical and pathological data. The limitations of this study are as follows: First, there have been many advances in the treatment of lung cancer over the past 15 years, and there are significant differences in survival rates between now and the past due to differences in treatment, which is one of the confounding factors in our study. Second, important factors associated with lung cancer prognosis, such as smoking history, cardiopulmonary function, postoperative complications, tumor markers and genetic information, could not be retrieved from the database in this study; and the time and site of recurrence, which are closely associated with lung cancer-specific death, were also unclear. Third, other inherent limitations of this retrospective study design include selection bias and information bias. Fourth, the prognosis based on SEER estimates for the US population may not accurately reflect that of other countries.

Despite the limitations mentioned above, the column line plots obtained in this study can be easily applied clinically and do not require any complex calculations, and they provide some reference for prognostic judgments and clinical decision-making during patient consultations. More importantly, the feasibility of column line plots in predicting CSS and OS were well verified in the present study, which also provides directions for future studies.

## Data availability statement

The raw data supporting the conclusions of this article will be made available by the authors, without undue reservation.

## Ethics statement

Ethical review and approval was not required for the study on human participants in accordance with the local legislation and institutional requirements. Written informed consent for participation was not required for this study in accordance with the national legislation and the institutional requirements.

## Author contributions

GY and XL: conceptualization. XL and YL: methodology, investigation, and supervision. YZ: software and formal analysis. RY and LZ: validation. ZW: resources, project administration, and funding acquisition. YZ, RY, and LZ: data curation. GY: writing—original draft preparation. XL: essay—review and editing. YZ and GY: visualization. All authors have read and agreed to the published version of the manuscript, contributed to the article, and approved the submitted version.

## Conflict of interest

Authors LZ and ZW were employed by Artificial Intelligence Laboratory, Pharnexcloud Digital Technology (Chengdu). The remaining authors declare that the research was conducted in the absence of any commercial or financial relationships that could be construed as a potential conflict of interest.

## Publisher's note

All claims expressed in this article are solely those of the authors and do not necessarily represent those of their affiliated organizations, or those of the publisher, the editors and the reviewers. Any product that may be evaluated in this article, or claim that may be made by its manufacturer, is not guaranteed or endorsed by the publisher.
